# A randomized, placebo-controlled trial of the BTK inhibitor zanubrutinib in hospitalized patients with COVID-19 respiratory distress: immune biomarker and clinical findings

**DOI:** 10.3389/fimmu.2024.1369619

**Published:** 2025-01-21

**Authors:** Steven P. Treon, Camille N. Kotton, David J. Park, Giorgia Moranzoni, Camilla K. Lemvigh, Joseph C. Gathe, Tilly A. Varughese, Christopher F. Barnett, Johnny M. Belenchia, Nina M. Clark, Charles M. Farber, Muhammad Bilal Abid, Gulrayz Ahmed, Christopher J. Patterson, Maria L. Guerrera, Jacob D. Soumerai, Vipheaviny A. Chea, Isabel P. Carulli, Jackson Southard, Shuqiang Li, Catherine J. Wu, Kenneth J. Livak, Eric Holmgren, Pil Kim, Carrie Shi, Holly Lin, Vanitha Ramakrishnan, Ying Ou, Scott Olszewski, Lars Rønn Olsen, Derin B. Keskin, Zachary R. Hunter, Christopher Tankersley, Todd Zimmerman, Binod Dhakal

**Affiliations:** ^1^ Dana-Farber Cancer Institute, Boston, MA, United States; ^2^ Massachusetts General Hospital, Boston, MA, United States; ^3^ Providence St. Jude Medical Center/Providence Medical Foundation, Fullerton, CA, United States; ^4^ Department of Health Technology, Technical University of Denmark, Kongens Lyngby, Denmark; ^5^ Therapeutic Concepts, PA, Houston, TX, United States; ^6^ Rutgers New Jersey Medical School, Newark, NJ, United States; ^7^ MedStar Washington Hospital Center, Washington, DC, United States; ^8^ Archbold Medical Center, Thomasville, GA, United States; ^9^ Loyola University Stritch School of Medicine, Chicago, IL, United States; ^10^ Atlantic Health System, Morristown, NJ, United States; ^11^ Medical College of Wisconsin, Milwaukee, WI, United States; ^12^ BeiGene USA, Inc., San Mateo, CA, United States; ^13^ Harvard Medical School, Boston, MA, United States; ^14^ Medical College of Wisconsin, Wauwatosa, WI, United States

**Keywords:** SARS-CoV-2, BTK, zanubrutinib, inflammatory mediators, serological response, single cell RNA analysis

## Abstract

**Background:**

Cytokine release triggered by a hyperactive immune response is thought to contribute to severe acute respiratory syndrome coronavirus 2019 (SARS-CoV-2)–related respiratory failure. Bruton tyrosine kinase (BTK) is involved in innate immunity, and BTK inhibitors block cytokine release. We assessed the next-generation BTK inhibitor zanubrutinib in SARS-CoV-2–infected patients with respiratory distress.

**Method:**

Cohort 1 had a prospective, randomized, double-blind, placebo-controlled design; cohort 2 had a single-arm design. Adults with SARS-CoV-2 requiring hospitalization (without mechanical ventilation) were randomized in cohort 1. Those on mechanical ventilation ≤24 hours were enrolled in cohort 2. Patients were randomized 1:1 to zanubrutinib 320 mg once daily or placebo (cohort 1), or received zanubrutinib 320 mg once daily (cohort 2). Co-primary endpoints were respiratory failure-free survival rate and time to return to breathing room air at 28 days. Corollary studies to assess zanubrutinib’s impact on immune response were performed.

**Results:**

Sixty-three patients in cohort 1 received zanubrutinib (n=30) or placebo (n=33), with median treatment duration of 8.5 and 7.0 days, respectively. The median treatment duration in cohort 2 (n=4) was 13 days; all discontinued treatment early. In cohort 1, respiratory failure-free survival and the estimated rates of not returning to breathing room air by day 28 were not significantly different between treatments. Importantly, serological response to coronavirus disease 2019 (COVID-19) was not impacted by zanubrutinib. Lower levels of granulocyte colony-stimulating factor, interleukin (IL)-10, monocyte chemoattractant protein-1, IL-4, and IL-13 were observed in zanubrutinib-treated patients. Moreover, single-cell transcriptome analysis showed significant downregulation of inflammatory mediators (IL-6, IL-8, macrophage colony-stimulating factor, macrophage inflammatory protein-1α, IL-1β) and signaling pathways (JAK1, STAT3, TYK2), and activation of gamma-delta T cells in zanubrutinib-treated patients.

**Conclusions:**

Marked reduction in inflammatory signaling with preserved SARS-CoV-2 serological response was observed in hospitalized patients with COVID-19 respiratory distress receiving zanubrutinib. Despite these immunological findings, zanubrutinib did not show improvement over placebo in clinical recovery from respiratory distress. Concurrent administration of steroids and antiviral therapy to most patients may have contributed to these results. Investigation of zanubrutinib may be warranted in other settings where cytokine release and immune cell exhaustion are important.

**Clinical Trial Registration:**

https://www.clinicaltrials.gov/study/NCT04382586, identifier NCT04382586.

## 1 Introduction

The coronavirus disease 2019 (COVID-19) pandemic, caused by severe acute respiratory syndrome coronavirus 2019 (SARS-CoV-2) (1), has been responsible for more than 650,000,000 infections and more than 6.6 million deaths worldwide (2). Respiratory failure is a leading cause of death in patients with COVID-19 (3, 4). SARS-CoV-2 triggers a hyperinflammatory response through the innate Toll-receptor signaling pathway by alveolar type II pneumocytes that bind to SARS-CoV-2 (5). This is amplified by resident macrophage and other immune cell activation through Toll-receptor signaling contributing to coronavirus-related pulmonary failure (6–10). Bruton tyrosine kinase (BTK) is a member of the Toll-receptor signaling pathway that is activated in response to viral and bacterial pathogens, including coronaviruses (11). Patients with SARS-CoV-2 infection exhibit higher levels of activated BTK in peripheral blood monocytes and release of the inflammatory cytokine interleukin (IL)-6 versus uninfected controls (12). The potential for BTK inhibitors to block inflammatory cytokine release was observed in patients with chronic lymphocytic leukemia (CLL), Waldenström macroglobulinemia (WM), and chronic graft-versus-host disease, and involved many of the proinflammatory and chemoattractant cytokines found elevated in the plasma of patients with SARS-CoV-2 (13–17).

Clinically, as reported by us and other investigators, patients with COVID-19–related respiratory distress have shown improved oxygenation following treatment with a BTK inhibitor (12, 18–22). These findings suggest that BTK inhibitors may block the hyperinflammatory response related to SARS-CoV-2. Zanubrutinib is a highly potent and selective covalent BTK inhibitor approved for the treatment of mantle cell lymphoma, marginal zone lymphoma, CLL/small lymphocytic lymphoma, and WM (23). Zanubrutinib demonstrates an improved safety profile, including a lower incidence of atrial fibrillation, in comparison to the first-generation BTK inhibitor ibrutinib (24, 25). This is particularly relevant because atrial fibrillation has been observed in up to 20% to 40% of patients with severe COVID-19 infections (26, 27). As such, we conducted a two-cohort, phase II study to evaluate the activity and safety of zanubrutinib in patients hospitalized with COVID-19 infection requiring supplemental oxygen due to respiratory distress. One cohort was a prospective, randomized, multicenter, double-blind, placebo-controlled trial, and the other was a single-arm study (BGB-3111-219).

## 2 Patients and methods

### 2.1 Clinical trial design

**Figure 1:**
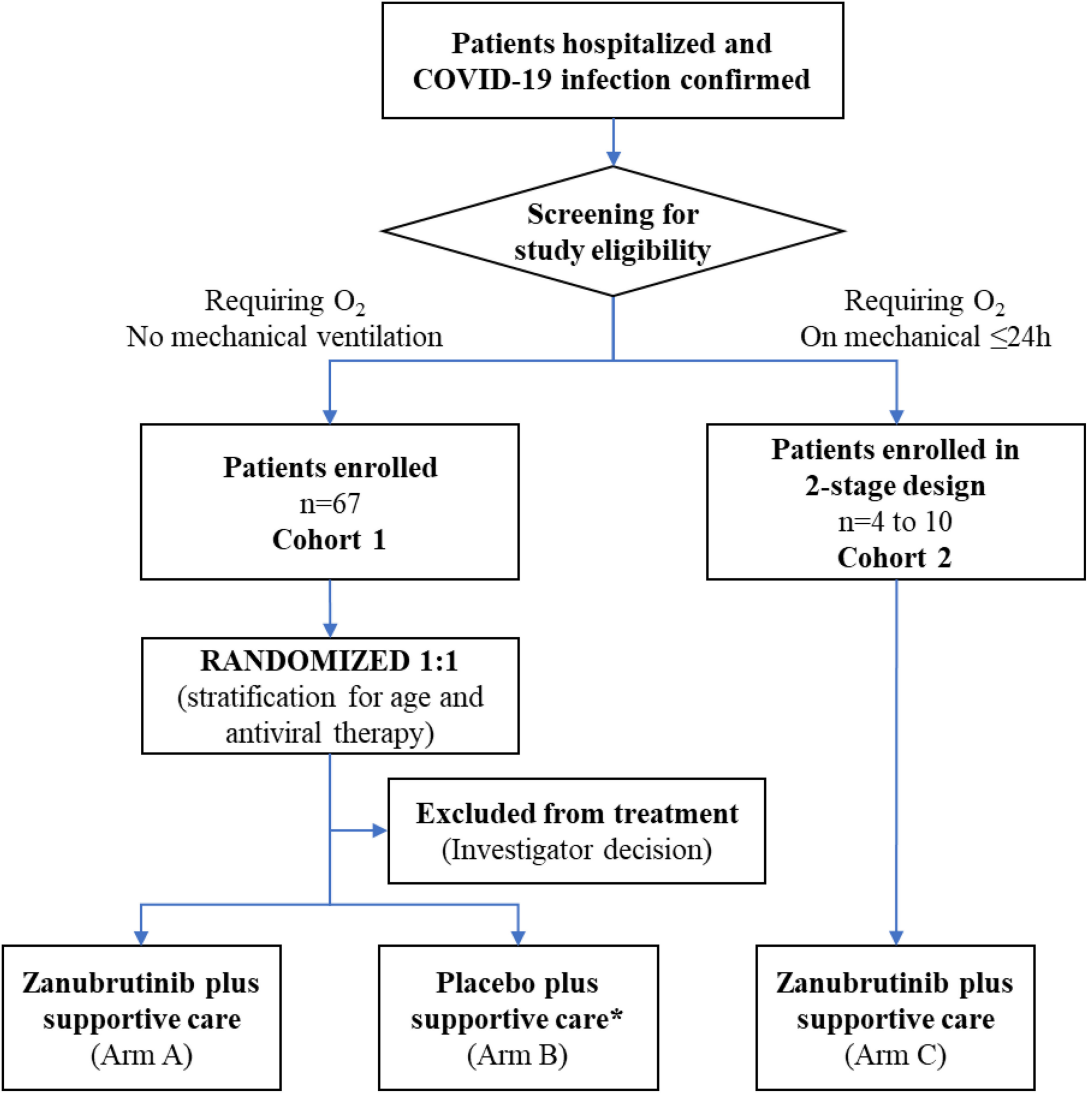
Patient flow through study. *Four patients randomized to the placebo arm were removed from the study before receiving study drug due to investigator decision, and were therefore excluded from the efficacy analysis.

BGB-3111-219 was a two-cohort study. Eligible adult patients in cohort 1 with a confirmed diagnosis by polymerase chain reaction test of COVID-19 infection and requiring supplemental oxygen for ≤96 hours were randomized 1:1 to oral zanubrutinib 320 mg once daily or matching placebo for a maximum of 28 days (**Figure 1**). Cohort 2 was an exploratory arm that evaluated the efficacy of zanubrutinib (320 mg once daily) in patients receiving mechanical ventilation for ≤24 hours. Patients in both cohorts were also required to have adequate organ and hematologic function and a C-reactive protein level of ≥8 mg/L at screening. Patients were recruited between July 6, 2020, and February 1, 2021. The study was performed following the Good Clinical Practice per International Conference on Harmonization Guideline E6 requirements under ethical principles in the Declaration of Helsinki. The study was developed under fast track with active FDA input. The protocol was approved by institutional review boards (IRB) at all participating institutions. Immunological studies were performed at the Dana Farber/Harvard Cancer Center under approved IRB protocols. All study participants (or designates) provided written consent before trial enrollment. Additional inclusion and exclusion criteria can be found in the **Supplementary Methods.**

The study’s co-primary endpoints were respiratory failure-free survival rate at 28 days and time to return to breathing room air. Efficacy endpoints were assessed for all randomized patients who received at least one dose of study drug, and safety endpoints were assessed in the intent-to-treat population. A safety review committee regularly reviewed unblinded safety data.

The full protocol can be found in the online supplement.

### 2.2 Biomarker studies

Serial evaluations of blood inflammatory cytokines and chemokines, single-cell transcriptome analysis, as well as immunoglobulin (Ig)M and IgG antibody titers to SARS-CoV-2, were performed as exploratory studies to identify clinically meaningful biomarkers for response activity, and to evaluate if zanubrutinib impacted humoral response to SARS-CoV-2. Patients who were medically unstable or inaccessible were excluded from corollary study sampling. Details of the biomarker analysis can be found in the **Supplementary Methods**.

### 2.3 Pharmacokinetic studies

In cohort 2, plasma samples from four treated patients were collected 2 hours after administration of a single zanubrutinib 320 mg dose, using open capsules prepared for nasogastric or feeding tube administration. The zanubrutinib capsules were gently squeezed before opening, to break up cakes, and the capsule contents were directly added to 50 mL of Sterile Water for Injection, USP. The zanubrutinib suspension was drawn into a syringe and administered via the nasogastric or feeding tube. An additional 50 mL of Sterile Water for Injection, USP, was drawn up and used to flush the syringe and the nasogastric or feeding tube. Plasma samples were analyzed using a validated liquid chromatography-tandem mass spectrometry method for the determination of zanubrutinib in K2 EDTA human plasma. The lower limit of quantitation was 1.0 ng/mL.

## 3 Results

Between July 6, 2020, and February 1, 2021, a total of 97 patients were screened. Of these, 63 were randomly assigned to receive zanubrutinib (n=30) or matching placebo (n=33) in cohort 1; four of the patients randomized to placebo did not receive study drug due to investigator decision and were excluded from the efficacy analysis. Therefore, in the efficacy analysis set, there were 29 patients in the placebo arm, and a total of 59 patients for cohort 1. Four patients were enrolled in cohort 2 (**Figure 1**). The baseline demographics for enrolled patients showed similar characteristics in both arms of cohort 1 and in cohort 2 as shown in **Table 1**.

**Table 1:** Baseline characteristics for patients in cohorts 1 and 2.

Characteristic	Cohort 1		Cohort 2
	Zanubrutinib	Placebo	Zanubrutinib
Patients randomized, n	30	33^a^	4
Age, years			
Mean (SD)	56.0 (12.51)	56.0 (13.30)	65.8 (14.15)
Median	58.0	57.0	67.0
Min, max	28, 81	27, 84	48, 81
Sex, n (%)			
Male	14 (46.7)	17 (51.5)	4 (100.0)
Female	16 (53.3)	16 (48.5)	0
Race, n (%)			
Asian	2 (6.7)	2 (6.1)	1 (25.0)
Black or African American	3 (10.0)	5 (15.2)	0
White	19 (63.3)	25 (75.8)	3 (75.0)
Other	1 (3.3)	1 (3.0)	0
Unknown	3 (10.0)	0	0
Not reported	2 (6.7)	0	0
BMI (kg/m^2^)			
n	30	28	4
Median	31.72	35.29	33.71
Min, max	19.5, 45.1	21.5, 60.3	19.4, 43.1
BMI ≥30	20 (66.7)	19 (57.6)	3 (75.0)
BMI <30	10 (33.3)	9 (27.3)	1 (25.0)
Missing	0	5 (15.2)	0
Clinical symptoms affecting >50% of patients at baseline, n (%)			
Cough	30 (100.0)	29 (87.9)	4 (100.0)
Shortness of breath	27 (90.0)	31 (93.9)	4 (100.0)
Fever	21 (70.0)	23 (69.7)	3 (75.0)
Fatigue	17 (56.7)	22 (66.7)	0
Efficacy analysis set^a^, n	30	29	4
Concomitant medications (in >50% of patients, n (%)			
Paracetamol	24 (80.0)	17 (58.6)	3 (75.0)
Remdesivir	22 (73.3)	19 (65.5)	1 (25.0)
Dexamethasone	21 (70.0)	20 (69.0)	3 (75.0)
Enoxaparin sodium	19 (63.3)	19 (65.5)	3 (75.0)
Other treatments received, n (%)			
Prior blood infusions for COVID-19	8 (26.7)	4 (13.8)	0
Plasma products^b^	8 (26.7)	7 (24.1)	0
IVIG	1 (1.03)	4 (13.8)	4 (100.0)

a Four patients randomized to the placebo arm did not receive study drug due to investigator decision; they were therefore excluded from the efficacy analysis set.

b Plasma products include COVID-19 convalescent plasma and cryoprecipitate. BMI, body mass index; COVID-19, coronavirus disease 2019; IVIG, intravenous immunoglobulin.

No patient in either cohort who received protocol therapy received a COVID-19 vaccination prior to study entry or during hospitalization. In cohort 1, among the 59 who received treatment, five (8.5%) patients received either an influenza or pneumococcal vaccine during hospitalization. Nearly half of these patients (44.4%) had three or more co-existing medical conditions at baseline that included hypertension (58.7%), diabetes mellitus (41.3%), asthma (14.3%), and chronic cardiac disease (12.7%); these were generally balanced between the treatment arms. All patients in cohort 1 were receiving supplemental oxygen at time of randomization, 47 (74.6%) with World Health Organization (WHO) grade 4 (oxygen by mask or nasal canula) and 16 (25.4%) with WHO grade 5 (non-invasive ventilation or high-flow oxygen) requirements. The median duration of treatment was 8.5 days in the zanubrutinib arm and 7.0 days in the placebo arm of cohort 1; and 13 days in cohort 2. Information on exposure and analysis sets can be found in the **Supplementary Data**.

### 3.1 Efficacy

A similar percentage of patients in the zanubrutinib and placebo arms had survived without respiratory failure at day 28 (90.0% vs 84.8% of patients, respectively; p=0.4099; **Table 2** and **Supplementary Figure 1A**). For the co-primary endpoint of time to return to breathing room air, there was no difference overall between the two arms (p=0.7619), with a similar percentage of patients in the zanubrutinib and placebo arms not returning to breathing room air at day 28 (26.7% vs 28.2% of patients, respectively; **Table 2** and **Supplementary Figure 1B**).

**Table 2:** Proportion of patients with respiratory failure-free survival and time to return to breathing room air for cohort 1 (intent-to-treat analysis set).

	Respiratory failure-free survival				Time to return to breathing room air, event-free rate^a^	
Day	Zanubrutinib n=30, n (%)	Placebo n=33, n (%)	Odds ratio (95% CI)	p-value^b^	Zanubrutinib n=30, % (95% CI)^c^	Placebo n=33, % (95% CI)^d^
7	28 (93.3)	29 (87.9)	1.93 (0.327-11.394)	0.3831	46.7 (28.4-63.0)	37.5 (21.3-53.7)
14	27 (90.0)	28 (84.8)	1.61 (0.349-7.391)	0.4099	36.7 (20.1-53.4)	31.3 (16.4-47.4)
21	27 (90.0)	28 (84.8)	1.61 (0.349-7.391)	0.4099	26.7 (12.6-43.0)	28.2 (14.1-44.1)
28^e^	27 (90.0)	28 (84.8)	1.61 (0.349-7.391)	0.4099	26.7 (12.6-43.0)	28.2 (14.1-44.1)

The hazard was calculated as total number of events divided by the total follow-up time (days); the hazard ratio and 95% CIs were estimated from a Cox regression model with placebo as reference group.

a Event-free rates were estimated by the Kaplan-Meier method with 95% CI estimated using Greenwood’s formula.

b The p-value was calculated from unstratified one-sided Fisher’s exact test.

c With a total follow-up of 364 days, 22 returned to breathing room air on or before day 28. Hazard 0.060; hazard ratio (95% CI) 0.918 (0.511-1.648); two-sided p-value=0.7619.

d With a total follow-up of 376 days, 23 returned to breathing room air on or before day 28. Hazard 0.061.

e Co-primary endpoint.

CI, confidence interval.

No statistically significant differences were observed between the zanubrutinib and placebo arms for any of the secondary endpoints, including the proportion of patients with respiratory failure or death, those discharged alive, those who died of any cause, those discharged from the intensive care unit alive, those with WHO 8-point scale improvement, number of days on mechanical ventilation, duration of hospitalization, or the use of assisted ventilation and oxygen therapy. Results for respiratory failure-free survival rate at day 28 and time to return to breathing room air for the per-protocol analysis set were similar to those in the intent-to-treat analysis set.

In cohort 1, 22 (73.3%) and 19 (65.5%) of placebo- and zanubrutinib-treated patients received remdesivir respectively. Moreover, 21 (70%) and 20 (69%) of placebo- and zanubrutinib-treated patients received dexamethasone, respectively. For cohort 2, only 1 (25%) patient received remdesivir, and 3 (75%) received dexamethasone (**Table 1**).

### 3.2 Safety

The proportion of patients with at least one treatment-emergent adverse event (TEAE) was similar in the zanubrutinib (63.3%) and placebo (69.0%) arms in cohort 1. All four patients in cohort 2 had at least one TEAE. Furthermore, the proportion of patients with at least one grade ≥3 TEAE was similar in the zanubrutinib (20.0%) and placebo (20.7%) arms in cohort 1 (**Table 3**). All four patients in cohort 2 had at least one grade ≥3 TEAE. The only grade ≥3 TEAEs reported in more than one patient in either treatment arm were respiratory failure (6.7%) in the zanubrutinib arm, and hypoxia (10.3%), hypoalbuminemia (6.9%), and respiratory failure (6.9%) in the placebo arm.

**Table 3:** Treatment-emergent adverse events (safety analysis set^a^).

TEAE, n (%)	Cohort 1		Cohort 2
	Zanubrutinib (n=30)	Placebo (n=29^a^)	Zanubrutinib (n=4)
At least one TEAE	19 (63.3)	20 (69.0)	4 (100.0)
At least one grade ≥3 TEAE	6 (20.0)	6 (20.7)	4 (100.0)
Leading to death	3 (10.0)	3 (10.3)	3 (75.0)
Leading to treatment discontinuation	2 (6.7)	1 (3.4)	0
Related TEAE	2 (6.7)	1 (3.4)	1 (25.0)
Most common TEAEs reported in ≥5% of patients in either cohort 1 or cohort 2^b^			
Bradycardia	5 (16.7)	0	0
Hypokalemia	3 (10.0)	1 (3.4)	0
Constipation	2 (6.7)	2 (6.9)	0
Diarrhea	2 (6.7)	1 (3.4)	0
Hyperglycemia	2 (6.7)	1 (3.4)	0
Nausea	2 (6.7)	0	0
Respiratory failure	2 (6.7)	2 (6.9)	0
Anemia	1 (3.3)	2 (6.9)	4 (100.0)
Headache	1 (3.3)	3 (10.3)	0
Hypoalbuminemia	1 (3.3)	2 (6.9)	3 (75.0)
Hypoxia	1 (3.3)	4 (13.8)	0
Hypomagnesemia	0	3 (10.3)	0
Pain in extremity	0	2 (6.9)	0
COVID-19 pneumonia	1 (3.3)	0	1 (25.0)
Acute respriatory distress syndrome	0	0	1 (25.0)
Hemorrhage intracranial	0	0	1 (25.0)
Hypernatremia	0	0	1 (25.0)
Septic shock	0	0	1 (25.0)

a Safety analysis set comprised all patients who received at least one dose of study drug. Four patients randomized to the placebo arm did not receive study drug due to investigator decision; they were therefore excluded from the safety analysis set.

b AEs classified based on MedDRA v23.0; patients with multiple events for a given PT were counted only once for each PT.

AE, adverse event; COVID-19, coronavirus disease 2019; MedDRA, Medical Dictionary for Regulatory Activities; PT, preferred term; TEAE, treatment-emergent adverse event.

In cohort 1, two (6.7%) patients in the zanubrutinib arm each had a fatal adverse event (AE) of respiratory failure unrelated to study drug. Two (6.7%) patients in the zanubrutinib arm had TEAEs assessed as related to treatment by the investigator: one had a non-serious grade 1 AE of rectal bleeding that resolved without change to study drug; and one had non-serious grade 1 AEs of diarrhea and muscle spasms without change to study drug, and these were ongoing at study end. One patient in the placebo arm had non-serious grade 1 AEs of hematuria and vaginal hemorrhage that resolved. In cohort 2, one patient had a serious fatal AE of intracranial hemorrhage, which started on study day 15 and was assessed by the investigator as being possibly related to zanubrutinib and a likely complication of COVID-19 pneumonia and concomitant anticoagulation.

A summary of TEAEs that led to death is presented in **Supplementary Table 1**. In cohort 1, three (10.0%) patients in the zanubrutinib arm and three (10.3%) in the placebo arm died during the study (one in each arm while on study treatment, and two in each arm within the 56-day safety follow-up period that occurred after end of treatment). In cohort 2, two (50.0%) patients died within the 56-day safety follow-up period following early discontinuation of the study drug due to investigator decision to pursue another line of therapy. In both cohorts, the primary reasons for death were related to progression of COVID-19 pneumonia, apart from the one patient in cohort 2 who died of intracranial hemorrhage while on study treatment.

### 3.3 Biomarkers

#### 3.3.1 SARS-CoV-2 antibody response

Since impairment of BTK can attenuate B-cell maturation and antibody response, we sought to clarify the impact of BTK inhibitor therapy on SARS-CoV-2 serological response (28). We therefore examined baseline and post-treatment serum levels for IgG antibodies to SARS.S1, SARS-CoV2.S1 protein and SARS-CoV2.spike trimer, SARS-CoV2-nucleocapsid, and SARS-CoV2.receptor-binding domain. As shown in **Supplementary Figure 2**, baseline values for all SARS-CoV-2 IgG antibody levels were higher in cohort 2 versus cohort 1 patients (all p<0.01). Following false discovery rate adjustment, all antibody levels remained significant (p<0.02). No statistical difference in baseline SARS-CoV-2 IgG antibody levels was observed between arms in cohort 1. We next examined changes in SARS-CoV-2 antibody over the first 7 days of study treatment. Patients in both arms of cohort 1 showed similar increases in SARS-CoV-2 IgG antibody responses at days 2 and 7 after randomization. Conversely, patients in cohort 2 showed a stable IgG antibody response to SARS-CoV-2 over the first 7 days (**Supplementary Table 2**). Specificity for SARS-CoV-2 viral antibody response was confirmed by control coronavirus antibody levels (COV.229E, CiV.HKU1, CoV.NL63, and CoV.OC43), which were similar at baseline and on days 2 and 7 across both arms of cohort 1 and cohort 2 (data not shown). Further to these studies, we sought to clarify relative changes in SARS-CoV-2 IgM and IgG antibody response by assessing serial SARS-CoV-2 spike trimer levels. At baseline, cohort 2 patients exhibited significantly higher IgM and IgG SARS-CoV-2 spike trimer levels versus those in cohort 1 (false discovery rate adjusted p-value <0.01; **Supplementary Table 2**). Levels of IgM and IgG SARS-CoV-2 spike trimer declined and remained stable over the course of 7 days. Conversely, both IgM and IgG SARS-CoV-2 spike trimer levels rose during the same period in cohort 1, without significant differences between arms (**Supplementary Table 2**).

#### 3.3.2 Inflammatory cytokine response

Since resident macrophage and other immune cell activation have been implicated in Toll-receptor mediated coronavirus-related pulmonary failure, we examined baseline and serial changes in chemokines, and proinflammatory and immune response cytokines in cohorts 1 and 2 (Refs 6-10; **Supplementary Table 3**). Patients in cohort 2 trended toward or showed significantly lower levels of granulocyte-macrophage colony-stimulating factor (p=0.09), IL-2 (p=0.029), IL-10 (p=0.06), and IL-17A (p=0.069), and higher levels of IL-6 (p=0.012) and IL-8 (p=0.009), relative to patients in cohort 1 at baseline (**Supplementary Table 3**). Baseline levels of cytokines showed no significant differences between the two arms of cohort 1 except for monocyte chemoattractant protein-1 (MCP-1), which was lower in zanubrutinib-treated patients. Following assigned study treatment, lower levels in the zanubrutinib arm compared with the placebo arm were observed for granulocyte colony-stimulating factor (G-CSF), IL-10, and MCP-1 on days 2 and 7 (p<0.05, both days), and for IL-4 and IL-13 on day 7 (p<0.05), in patients receiving zanubrutinib versus placebo (**Supplementary Table 4**). Following false discovery rate correction, these findings were statistically unremarkable.

#### 3.3.3 Single-cell RNA analysis

To differentiate immune cell contributions for inflammatory cytokine and chemokine changes related to zanubrutinib, we performed single cell RNA analysis in patients on cohort 1 (29). This exploratory analysis was based on peripheral blood mononuclear cell samples (**Supplementary Figure 3**) collected from six and seven patients randomized to the placebo and zanubrutinib arms of cohort 1, respectively, and focused on cytokines and chemokines implicated in SARS-COV-2 pathophysiology (10). At 2 days after zanubrutinib treatment, a significant downregulation was observed compared to pre-treatment in proinflammatory mediators IL-6, Janus kinase (JAK) 1, signal transducer and activator of transcription (STAT) 3, macrophage colony-stimulating factor (M-CSF), macrophage inflammatory protein (MIP)-1α, and IL-1β (**Table 4**; **Supplementary Table 5**). Importantly, IL-8, a major mediator of systemic and pulmonary inflammatory response and a putative biomarker, along with IL-6, for COVID-19 severity, was significantly downregulated in zanubrutinib-treated patients (10, 30). Tyrosine kinase 2, the overexpression of which was associated with life-threatening COVID-19 (31), was significantly downregulated in many cell types in zanubrutinib-treated patients, including CD4+ and CD8+ effector memory T cells, CD4+ central memory T cells, and memory B cells.

**Table 4:** Observations in the single-cell RNA analysis in samples from patients treated with zanubrutinib.

Cytokine/chemokine	Role	Cell type	Effect at day 2	p-value^a^
IL-6	Inflammatory mediator	Naive B cells and memory B cells	Downregulated	0.030
JAK1	Important for IL-6-related inflammatory signaling	CD14-expressing monocytes	Downregulated	0.007
STAT3	Important for IL-6-related inflammatory signaling	CD14-expressing monocytes	Downregulated	0.046
CSF1/ M-CSF	Regulates the production, differentiation, and migration of monocytes	CD14-expressing monocytes	Downregulated	0.029
MIP-1α	Promotes inflammation and chemokinesis	CD14-expressing monocytes, platelets, CD4 effector memory T cells, and plasmablasts	Downregulated	0.001-0.007
IL-1β	Promotes T-cell activation and cytokine release, as well as B-cell activation and humoral response	CD14-expressing monocytes	Downregulated	0.001
IL-8	Major mediator of systemic and pulmonary inflammatory response, and a putative biomarker along with IL-6 for COVID-19 severity (10, 27)	CD14 monocytes	Downregulated	0.009
TYK2	Promulgates cytokine signaling by activation of STAT family members	CD4 and CD8 effector memory T cells, CD4 central memory T cells, and memory B cells	Downregulated	0.016-0.038
IFNAR1	Heterodimeric cytokine receptor partner of TYK2 (28)	CD14-expressing monocytes	Downregulated	0.035
CXCR4	Triggers MAPK/ERK signaling as well as cell migration. Implicated in cytokine-mediated acute lung injury (29)	CD14-expressing monocytes, various CD4 and CD8 T-cell subsets, γδ-T cells, NK cells, memory B cells, and plasmablasts	Downregulated	0.000-0.030
IFN-γ	γδ-T cells, with a key role in early detection of viral infections, can suppress viral replication, as well as recruit and activate other immune cells such as macrophages, T cells, and NK cells through release of IFN-γ (30)	γδ- T cells	Upregulated	0.012

a Based on comparisons between baseline and day 2.

γδ, gamma delta; COVID-19, coronavirus disease 2019; CXCR, C-X-C chemokine receptor; CSF, colony-stimulating factor; ERK, extracellular signal-regulated kinase; IFN, interferon; IFNAR, interferon alpha and beta receptor; IL, interleukin; JAK, Janus kinase; MAP, mitogen-activated protein; M-CSF, macrophage colony-stimulating factor; MIP, macrophage inflammatory protein; NK, natural killer; STAT, signal transducer and activator of transcription; TYK, tyrosine kinase.

Unexpectedly, we observed a significant upregulation in interferon-gamma (IFN)-γ in gamma-delta (γδ)-T cells in zanubrutinib-treated patients, indicative of their activation. γδ-T cells have a key role in early detection of viral infections and can suppress viral replication, as well as recruit and activate other immune cells such as macrophages, T cells, and natural killer cells through release of IFN-γ (32). In contrast to zanubrutinib-treated patients, those in the placebo arm showed a slight downregulation in IFN-γ, indicating an unexpected role for zanubrutinib in triggering γδ-T-cell activation. Lastly, in contrast to zanubrutinib-treated patients, those in the placebo arm also showed upregulated levels of M-CSF in CD4+ effector memory T cells, as well as macrophage inflammatory protein (MIP)-1α and tumor necrosis factor (TNF)-α in CD14-expressing monocytes.

### 3.4 Pharmacokinetics

To evaluate the pharmacokinetics of zanubrutinib when administered via a nasogastric or feeding tube (cohort 2), zanubrutinib concentrations in plasma from individual patients were assessed 2 hours after dose administration, which corresponds to approximately the occurrence of peak plasma concentrations following oral administration of zanubrutinib. The data were available from four patients in cohort 2 who were on mechanical ventilation for ≤24 hours. Zanubrutinib was present at measurable concentrations of 14.9, 21.2, 31.3, and 92.7 ng/mL, respectively, at 2 hours after dose administration via a nasogastric or feeding tube.

## 4 Discussion

We investigated the activity of zanubrutinib in a prospective study that included a randomized, double-blind cohort, to add to the evidence from preclinical and clinical studies that supports a potential role for BTK inhibitors to block SARS-CoV-2–related respiratory distress by suppressing pulmonary inflammatory responses (8, 10, 12, 22, 33). Our findings showed that zanubrutinib did not statistically differ from placebo for either respiratory failure-free survival rate or time to return to breathing room air at day 28.

A lack of meaningful clinical benefit was also reported in a prospective, placebo-controlled, randomized study of ibrutinib in 46 patients with COVID-19 who were hospitalized with hypoxia (34). Similar to our study, most patients received concomitant remdesivir and/or dexamethasone. The primary endpoint for the proportion of patients alive and without respiratory failure at day 28 was not met in that study. The addition of the BTK inhibitor acalabrutinib to best supportive care in patients with SARS-CoV-2 respiratory symptoms also failed to increase the proportion of patients who remained alive and free of respiratory failure in the two phase II CALAVI trials (NCT04380688 and NCT04346199); the formal publications of these two studies are awaited. Importantly, the TEAEs for zanubrutinib were similar to placebo in our study, thereby providing continued evidence for the safety profile of zanubrutinib. One patient in cohort 2 died of intracranial hemorrhage that was assessed as possibly related to the study drug, SARS-CoV-2 infection, and/or concomitant anticoagulation. Intracranial hemorrhage is a known complication of COVID-19, with an incidence of 8% among hospitalized patients (35, 36).

Most patients in the current study and the ibrutinib study (34) received steroids and antiviral therapy, which may have confounded the ability to observe differences between the active treatment and placebo arms with respect to the primary and secondary endpoints. Regarding biomarker findings, no differences in SARS-CoV-2 IgM or IgG antibody response were observed between patients in the zanubrutinib and placebo arms of cohort 1, whereas SARS-CoV-2 IgG antibody levels were higher in cohort 2 versus cohort 1 patients. Similar findings were reported by Roltgen et al. (37) who observed higher levels of SARS-CoV-2 IgG antibody response among those patients with more severe illness that required intensive care. A poor response to the COVID-19 vaccination has been observed in patients with CLL and WM receiving BTK inhibitors, although as many as half of patients with CLL and WM who were treatment-naive also lacked serological response to vaccination (38, 39). Many of these patients show a paucity of immune effector response. Placed into context with the findings from the current study, the contribution of BTK inhibitors alone is unlikely to be responsible for a blunted vaccine response in patients with CLL or WM. Treatment with a BTK inhibitor before SARS-CoV-2 vaccination, as well as qualitative differences for immune stimulation between vaccination and infection, may also have contributed to the blunted vaccination responses observed in patients with CLL or WM on BTK inhibitors.

The key circulating cytokines implicated in COVID-19 pathology were also noteworthy (9, 10, 40, 41). Patients who received zanubrutinib displayed lower levels for G-CSF, IL-10, and MCP-1 on days 2 and 7, as well as IL-4 and IL-13 on day 7. However, MCP-1 levels in zanubrutinib-treated patients in cohort 1 were significantly lower at baseline. Surprisingly, other cytokines elevated in COVID-19 patients such as C-X-C motif chemokine ligand 10, IL-6, MIP-1α, and TNF-α, which are downregulated in patients with certain B-cell malignancies and graft-versus-host disease treated with BTK inhibitors, showed no differences between zanubrutinib- and placebo-treated patients in cytokine studies (13–16). The lack of impact of zanubrutinib- versus placebo-treated patients on these cytokines may have reflected the concurrent use of steroids, which most patients in this study received. In addition, the cell of origin and signaling cascades for C-X-C motif chemokine ligand 10, IL-6, MIP-1α, and TNF-α production in COVID-19 patients may differ from those with B-cell malignancies and graft-versus-host disease; therefore, the effects of BTK inhibitors may differ in these patient groups.

Particularly relevant were single-cell RNA studies that depicted the impact of zanubrutinib treatment on cytokines, chemokines, and relevant receptor signaling pathways in circulating immune cell populations. These studies, though exploratory in nature, revealed novel insights into the activity of zanubrutinib that may also be relevant in other disease settings. CD14-expressing monocytes showed significant downregulation in multiple inflammatory cytokines including M-CSF, MIP-1α, MIP-3α, IL-1β, IL-8, and multiple members of the JAK-STAT pathway that contribute to the inflammatory response.

Microenvironmental support of malignant cells by monocytes through release of inflammatory proteins has been reported in CLL and WM, and their inhibition by zanubrutinib and other BTK inhibitors may contribute to their mechanism of action (42, 43). Downregulation of JAK-STAT pathway members was also observed among various CD4 and CD8 T-cell populations. Notable was the downregulation of CXCR4 in response to zanubrutinib across many immune cell populations, a known consequence of BTK inhibitors in B-cell malignancies (44). CXCR4 has been implicated in acute lung injury by triggering inflammatory response through MAPK and nuclear factor-kB (45).

An unexpected finding of our study was the upregulation of IFN-γ in γδ-T cells, denoting activation of this specialized population of immune effector cells. γδ-T cells mediate direct pathogen cytotoxic activity, but also have antitumor activity that is independent of human leukocyte antigen recognition (46). Although studies addressing the impact of BTK inhibitors on γδ-T cells are limited, an inhibitory effect of ibrutinib on γδ-T cell activity has been reported, which contrasts with the findings in this study with zanubrutinib (46, 47). Such differences may be related to the off-target inhibition of ITK by ibrutinib, which is not shared by zanubrutinib (48, 49).

Our findings may also be relevant to the potential use of zanubrutinib as an adjunct to cellular immune-based treatments. Cytokine release syndrome (CRS) is a common adverse effect associated with cellular immunotherapies including chimeric antigen receptor T-cell and bispecific antibody therapies (50). Many of the primary and secondary inflammatory cytokines and signaling pathways involved in CRS were markedly abrogated by zanubrutinib, including IL-1β, IL-6, IL-8, G-CSF, and MIP-1α (50–52). Tocilizumab is used to block IL-6 in the prophylaxis and treatment of CRS, demonstrating the importance of agents directed at cytokine suppression with cellular immunotherapies (52–55). Zanubrutinib may offer an advantage over IL-6 antibodies by more broadly blocking inflammatory pathways and signaling cascades relevant to CRS. In addition, myeloid derived suppressor cells, including monocytic myeloid derived suppressor cells, play a fundamental role in tumor propagated T-cell exhaustion through elaboration of inflammatory mediators that include IL-6 (56). Lastly, our unexpected finding of γδ-T cell activation following zanubrutinib treatment represents the first report of such activity, and may be relevant to the development of chimeric antigen receptor γδ-T cells and/or bispecific antibodies.

In summary, we conducted a prospective, randomized, multicenter, double-blind study to examine the impact of zanubrutinib versus placebo in hospitalized COVID-19 patients in respiratory distress. We observed a marked reduction in inflammatory cytokine signaling along with preserved serological response in those receiving zanubrutinib. Despite the immunological impact, zanubrutinib did not show improvement over placebo in clinical recovery from respiratory distress. Concurrent administration of steroids and antiviral therapy to most patients in this study may have contributed to these results. The data from this prospective, double-blind study may support the investigation of zanubrutinib in other settings where cytokine release and immune cell exhaustion are important.

## Data Availability

Datasets are available on request. The raw data supporting the conclusions of this article will be made available by the authors, without undue reservation.

## In memoriam

The study is dedicated to the memory of Francisco Marty, MD, Associate Professor of Medicine at Harvard Medical School, and senior physician in the Division of Infectious Diseases at the Brigham and Women’s Hospital (Boston, Massachusetts). Dr Marty was a masterful physician, a dedicated researcher, and a superb teacher. He spearheaded multiple trials devoted to COVID-19, including this study. His expertise greatly added to the development of this study, and his devotion to the care of hospitalized patients with COVID-19 was exemplary. His death during the study following a tragic accident was a great loss to all who knew him. May this study also serve as an enduring memorial to his impactful life.
