# Supplementary material for: A randomized, placebo-controlled trial of the BTK inhibitor zanubrutinib in hospitalized patients with COVID-19 respiratory distress: immune biomarker and clinical findings
Source: Front Immunol. 2025 Jan 21;15:1369619. doi: 10.3389/fimmu.2024.1369619 (PMC11791645; doi:10.3389/fimmu.2024.1369619)
Supplement: Supplementary file 1 [file DataSheet1.docx]

**Supplementary Material**

**Contents**

[Supplementary Methods 2](#_Toc152243824)

[**Clinical Trial Design 2**](#_Toc152243825)

[**Sample Size and Statistical Considerations 3**](#_Toc152243826)

[**Analysis Sets 4**](#_Toc152243827)

[**Biomarker Studies 5**](#_Toc152243828)

[Supplementary Results 7](#_Toc152243829)

[**Exposure 7**](#_Toc152243830)

[**Supplementary Figure 1. Kaplan-Meier Plot for Respiratory Failure-Free Survival (A) and Time to Return to Breathing Room Air (B) for Cohort 1. 8**](#_Toc152243831)

[**Supplementary Figure 2. Baseline Levels for Antibodies for Cohort 1 and Cohort 2 Patients to SARS-CoV-2 Antigenic Determinants 9**](#_Toc152243832)

[**Supplementary Figure 3. UMAP Cell Type Clusters. 10**](#_Toc152243833)

[**Supplementary Table 1: Treatment-Emergent Adverse Events Leading to Death by System Organ Class and Preferred Term. 11**](#_Toc152243834)

[**Supplementary Table 2. Serial Antibody Responses for Patients in Cohorts 1 and 2. 13**](#_Toc152243835)

[**Supplementary Table 3. Baseline Cytokine and Chemokine Levels for Patients in Cohort 1 and 2. 15**](#_Toc152243836)

[**Supplementary Table 4. Baseline and Serial Cytokine and Chemokine Levels for Patients in Cohort 1 and 2. 17**](#_Toc152243837)

[**Supplementary Table 5. Findings From Single-Cell Transcriptome Studies for Cohort 1 Patients. 22**](#_Toc152243838)

[Supplementary References 26](#_Toc152243839)

# Supplementary Methods

## **Clinical Trial Design**

BGB-3111-219 study was conducted at 11 critical care hospitals in the United States. In cohort 1, eligible patients were adults (aged ≥18 years) with a diagnosis of a COVID-19 infection confirmed by polymerase chain reaction test within 10 days and requiring supplemental oxygen for no more than 4 days prior to screening. Patients were also required to have adequate organ and hematologic function and a C-reactive protein level of 8 mg/L or more at screening. Prior Bruton tyrosine kinase (BTK) inhibitor treatment, use of anti-CD20 monoclonal antibody therapy, and immune checkpoint inhibitors were exclusionary. Patients with asymptomatic arrhythmias (e.g., non-sustained ventricular tachycardia, bradycardia heart rate less than 50 beats/minute, or atrioventricular block, or any other atrial or ventricular arrhythmia) and/or known history of ejection fraction less than 40% were excluded.

Randomization was performed with a central interactive response system and permuted block randomization stratified by age and receipt of antiviral therapy.

Patients in cohort 1 were randomized to receive zanubrutinib or matched placebo in a double-blind fashion. Investigators, study site staff, participating cohort 1 patients, and the sponsor core study team were masked to treatment assignment during and after treatment assignment. All patients enrolled into cohort 2 received open-label zanubrutinib. Per protocol, investigators could request a patient’s treatment assignment in emergency cases. Laboratory data were also blinded before the primary analysis, including safety laboratory results, C-reactive protein, and biomarker results (immunoglobulin [Ig]G and IgM antibody titers, inflammatory and chemoattractant cytokines, transcriptome profiling). At the conclusion of the study the sponsor unblinded the study in order to perform the final study analyses. Study drugs (zanubrutinib and placebo) were supplied by BeiGene Ltd in identical bottles and packaging, labeling, schedule of administration, and appearance. Supportive care therapy was permitted including use of antiviral treatment, intravenous immunoglobulin, convalescent plasma, dexamethasone, and anticoagulation therapy. The maximum duration of study drug treatment was 28 days, with provisions for early discontinuation of clinical benefit if patients were considered clinically stable on room air for ≥24 hours with an oxygen saturation of at least 94%. Following the 28-day treatment period, patients had two required follow-up safety assessments at 30 days and 56 days post-treatment.

## **Sample Size and Statistical Considerations**

At the time of study initiation, there was no standard of care in place for COVID-19. Sample size was therefore informed by the respiratory failure-free survival and death rates of the standard-of-care arm of the lopinavir-ritonavir COVID-19 study (1) (see protocol; available from: https://classic.clinicaltrials.gov/ProvidedDocs/86/NCT04382586/Prot_000.pdf ). The study was predicated on enrolling 46 to 52 patients, with 42 patients in cohort 1 randomized to either zanubrutinib and supportive care (arm A) or placebo and supportive care (arm B). Cohort 2 was exploratory in nature with enrollment of 4 to 10 patients, with all patients treated with zanubrutinib and supportive care. Assuming a 10% dropout prior to day 28, unrelated to efficacy, 38 patients in total in cohort 1 would provide approximately 81% power to detect an increase in the respiratory failure-free survival rate from 70% to 95% under a z-test for proportions with 1-sided type I error of 0.10. Moreover, it would also provide 77% power for detecting an increase from 80% to 99% and 64% power for detecting an increase from 85% to 99% both with 1-sided type I error of 0.10. For cohort 2, assumptions were based on the study of Richardson et al. (2), who reported a death rate for patients on ventilators of 88.1% (282/320 patients). The 95% confidence interval for the death rate is 84.6-91.6, and the associated 95% confidence interval for the discharged alive rate is 8.4-15.4. A Simon’s 2-stage design was used to construct a test of the null hypothesis that the discharge rate for patients receiving zanubrutinib and supportive care is 15% against the alternative that the discharge rate is 45%. Details are provided in the protocol (see protocol; available from: https://classic.clinicaltrials.gov/ProvidedDocs/86/NCT04382586/Prot_000.pdf). In the first stage, four patients were to be enrolled. If at least one patient from cohort 2 was evaluated as attaining a World Health Organization ordinal scale of ≤3 or discharged alive, then six additional patients would be enrolled for a total of 10 patients. Patients whose discharged/survival status was unknown would not be counted as discharged alive. The null hypothesis that the discharged alive rate is 15% would be rejected if four or more patients discharged alive are observed in 10 patients. This design yields a 1-sided type I error rate of 0.0469 and power of 0.8066 when the true discharged alive rate is 45%.

## **Analysis Sets**

In cohort 1, all randomized patients in the zanubrutinib arm were included in the intent-to-treat (ITT), safety, and per-protocol analysis sets. All randomized patients in the placebo arm were included in the ITT analysis set, and the four patients who did not receive treatment were excluded from the safety and per-protocol analysis sets. In cohort 2, the four treated patients were included in the efficacy, safety, and pharmacokinetics analysis sets.

## **Biomarker Studies**

Viral inactivation was performed by treating the samples with TritonX-100 (SIGMA #93443) at a final concentration of 1%, vortexed and incubated at room temperature for 2 hours. Samples were then centrifuged at 10,000 x g for 10 min and the supernatants were used for enzyme-linked immunoabsorbent assay (ELISA). Severe acute respiratory syndrome coronavirus 2 (SARS-CoV-2) antibody measurements were determined by ELISA (IgG ELISA Kit, ThermoFisher Scientific, Waltham, MA) according to the manufacturer’s guidance. Cytokine and chemokine levels were determined using manufacturer’s directions on the ProcartaPlex platform: Human Cytokine Storm Panel 21-plex; Human IP-10 Simplex; Human HS; Mix & Match 2-plex; IL-6 Human Simplex Kit, High Sensitivity; and IL-8 Human Simplex Kit, High Sensitivity (ThermoFisher Scientific). These assays were evaluated on the Luminex MAGPIXâ instrument and analyzed with the xPONENT software. All assays were acquired on the SpectraMax i3x instrument (Molecular Devices, San Jose, CA) and analyzed with the SoftMax Pro software.

For single-cell transcriptome analysis, blood samples were processed in a BSL2+ cell culture facility at TIGL-DFCI. Peripheral blood mononuclear cells (PBMCs) were isolated by Ficoll-Paque centrifugation. PBMCs were frozen in 10% dimethyl sulfoxide 90% fetal bovine serum freezing media. Serum and PBMC samples were frozen and stored in liquid nitrogen until experimentation. PBMCs were thawed, and live single cells were isolated using a live cell isolation kit (Miltenyi Biotech, Waltham, MA). The cells were washed and resuspended in phosphate-buffered saline with 0.04% bovine serum albumin at a cell concentration of 1000 cells/µL. About 17,000 viable cells were loaded onto a 10× Genomics Chromium^TM^ instrument (10x Genomics, Pleasanton, CA) according to the manufacturer’s recommendations. The single-cell RNA sequencing (scRNAseq) libraries were processed using Chromium Next GEM Single Cell 5' Kit v2 (10x Genomics). Quality controls for amplified cDNA libraries and final sequencing libraries were performed using Bioanalyzer High Sensitivity DNA Kit (Agilent Technologies, Lexington, MA). The sequencing libraries for scRNAseq were normalized to 4nM concentration and pooled. The pooled sequencing libraries were sequenced on Illumina NovaSeq S4 300 cycle platform at the Broad Institute. The sequencing parameters were: Read 1 of 26bp, Read 2 of 90bp, Index 1 of 10bp, and Index 2 of 10bp. The sequencing data were demultiplexed and aligned to GRCh38-2020 using Cell Ranger (version 5.0.0) pipeline (10x Genomics). Samples were then analyzed using Seurat (v. 4.0.4 and 4.1.0). To correct for potential batch effects, data were integrated as described by Stuart et al. (3). Following integration, the data were scaled, a principal component analysis performed, and cells clustered according to the standard workflow of Seurat. Cell types were annotated by reference mapping using the cellular indexing of transcriptomes and epitopes by sequencing (CITE-seq) dataset consisting of 162,000 PBMCs with 228 antibody measurements (4). These were further validated by detection of marker genes for each cluster. Differential gene expression analyses were performed using DESeq2 (v. 1.34.0). This was done by generating a pseudo-bulk count matrix for each cell type. Differences in cell type proportions between zanubrutinib and placebo were tested for each time point using both propeller from speckle and a Wilcoxon test (5).

# Supplementary Results

## **Exposure**

The maximum study drug treatment duration specified per protocol was 28 days; all patients were treated for ≤28 days except one patient who was treated for 29 days. The median duration of treatment was 8.5 days in the zanubrutinib arm and 7.0 days in the placebo arm for cohort 1, and 13 days in cohort 2. Dose interruptions were low in cohort 1 with one patient in the zanubrutinib arm experiencing a non-serious grade 1 adverse event (AE) of nausea that resolved and was considered not related to study drug. One patient in the placebo arm had a non-serious AE of grade 1 vaginal hemorrhage that resolved and was assessed as related to treatment. In cohort 1, one patient in each arm had a single dose level reduction; one patient in the zanubrutinib arm received only a partial dose due to clinical deterioration and one patient in the placebo arm received a 50% reduction due to concomitant treatment with amlodipine and atenolol for hypertension that was ongoing from baseline. No dose interruptions or reductions occurred in patients enrolled into cohort 2.

## **Supplementary Figure 1. Kaplan-Meier Plot for Respiratory Failure-Free Survival (A) and Time to Return to Breathing Room Air (B) for Cohort 1.**


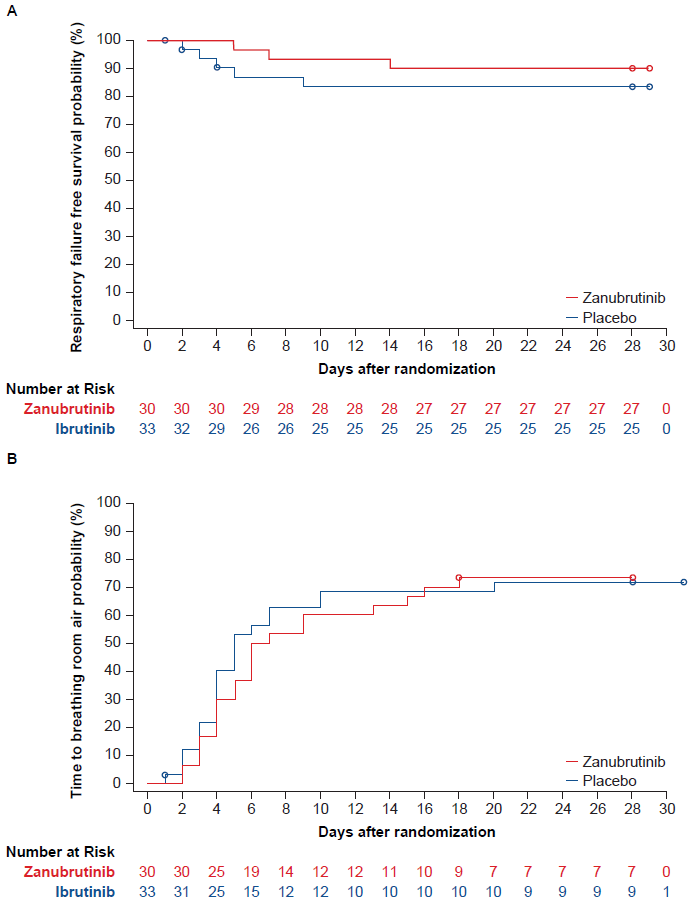


## **Supplementary Figure 2. Baseline Levels for Antibodies for Cohort 1 and Cohort 2 Patients to SARS-CoV-2 Antigenic Determinants**


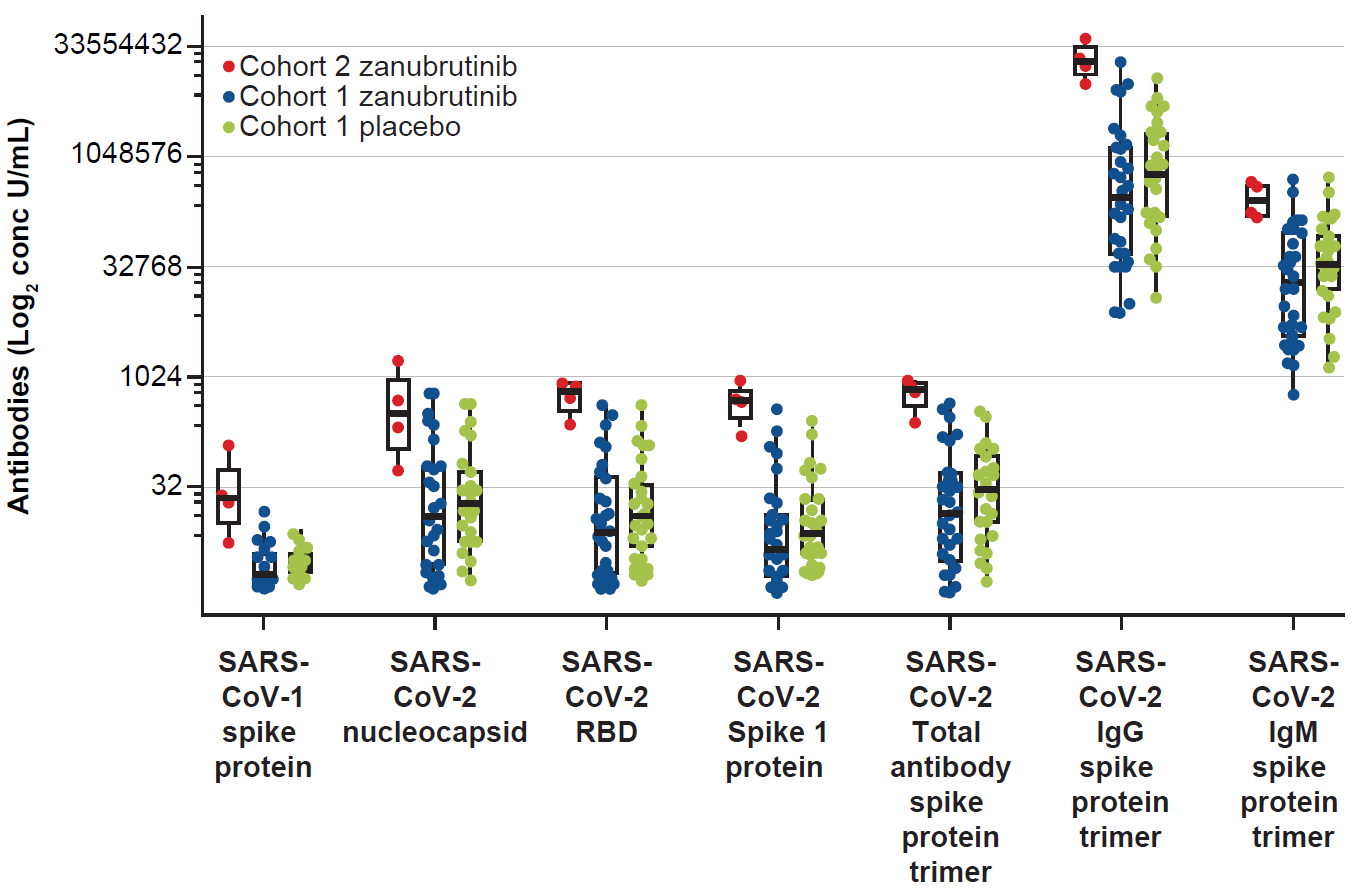


Ig, immunoglobulin; RBD, receptor binding domain; SARS-CoV 1/2, severe acute respiratory syndrome coronavirus 1/2.

## **Supplementary Figure 3. UMAP Cell Type Clusters.**


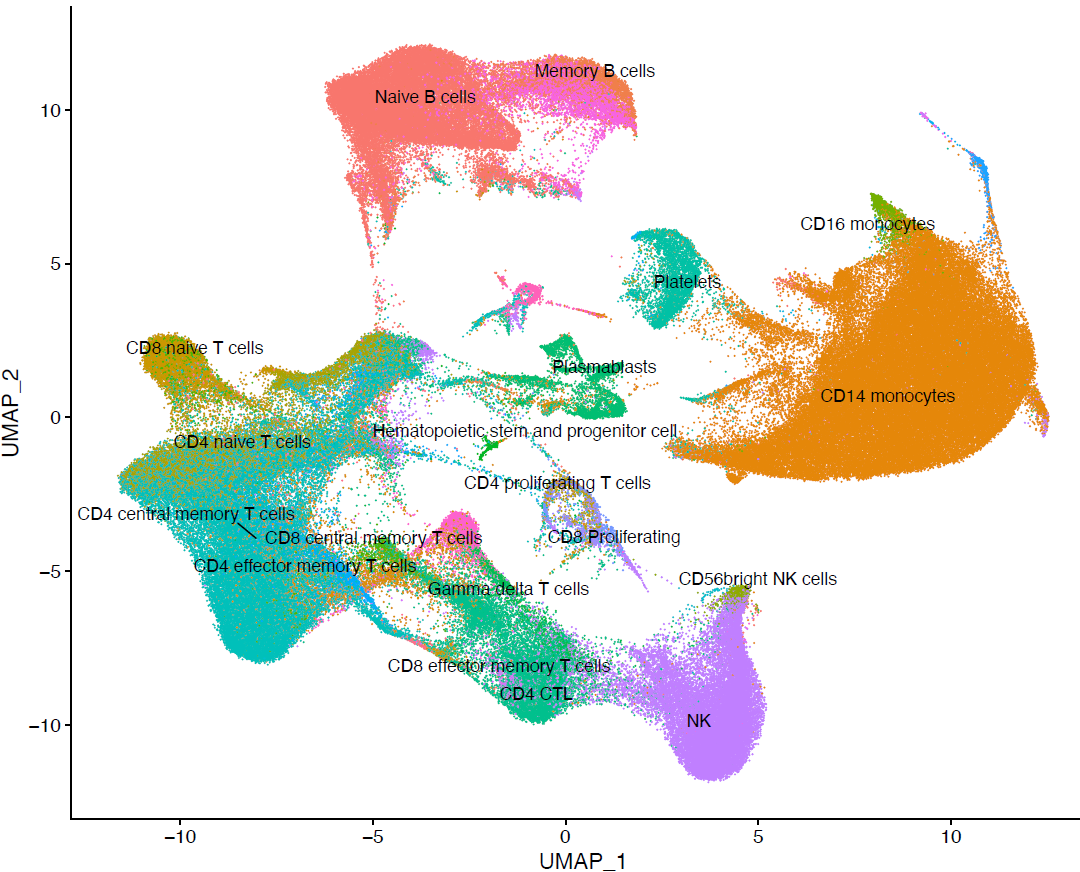


UMAP, uniform manifold approximation and projection.

## **Supplementary Table 1: Treatment-Emergent Adverse Events Leading to Death by System Organ Class and Preferred Term.**

| **Primary death reason^a^** | **Cohort 1** | | **Cohort 2** |
| --- | --- | --- | --- |
|  | **Zanubrutinib**  **(n=30)**  **n (%)** | **Placebo^b^ (n=29) n (%)** | **Zanubrutinib (n=4)**  **n (%)** |
| Patients with ≥1 TEAE leading to death | 3 (10.0)^c^ | 3 (10.3)^d^ | 3 (75.0)^e^ |
| Respiratory, thoracic, and mediastinal disorders | 2 (6.7) | 2 (6.9) | 1 (25.0) |
| Respiratory failure | 2 (6.7) | 1 (3.4) | 0 |
| Acute respiratory distress syndrome | 0 | 0 | 1 (25.0) |
| Respiratory distress | 0 | 1 (3.4) | 0 |
| Infections and infestations | 1 (3.3) | 0 | 1 (25.0) |
| COVID-19 pneumonia | 1 (3.3) | 0 | 1 (25.0) |
| Septic shock | 0 | 0 | 1 (25.0) |
| Renal and urinary disorders | 1 (3.3) | 0 | 1 (25.0) |
| Acute kidney injury | 1 (3.3) | 0 | 1 (25.0) |
| Vascular disorders | 1 (3.3) | 0 | 0 |
| Distributive shock | 1 (3.3) | 0 | 0 |
| Cardiac disorders | 0 | 1 (3.4) | 0 |
| Right ventricular failure | 0 | 1 (3.4) | 0 |
| Nervous system disorders | 0 | 0 | 1 (25.0) |
| Hemorrhage intracranial | 0 | 0 | 1 (25.0) |

.

^a^AEs classified based on MedDRA v. 23.0. Safety analysis set is defined as all patients who received at least 1 dose of study drug. Patients with multiple events for a given PT and SOC were counted only once for each PT and SOC, respectively.

^b^Four patients randomized to placebo were removed from the study before receiving study drug due to investigator decision, and were therefore excluded from the efficacy analysis.

^c^For the three patients in the zanubrutinib arm who died, one had a fatal AE of respiratory failure; one had fatal AEs of respiratory failure, distributive shock, and acute kidney injury; and one had a fatal AE of COVID-19 pneumonia.

^d^In the placebo group, two patients died due to COVID-19‒related respiratory failure and one due to right heart failure from progression of COVID-19

^e^In cohort 2, one patient had a fatal AE of acute respiratory distress syndrome; one patient had a fatal AE of intracranial hemorrhage; and one patient had fatal AEs of acute kidney injury, septic shock, and COVID-19 pneumonia.

AE, adverse event; MedDRA, Medical Dictionary for Regulatory Activities; PT, preferred term; SOC, system organ class; TEAE, treatment-emergent adverse event

## **Supplementary Table 2. Serial Antibody Responses for Patients in Cohorts 1 and 2.**

Baseline, day 2, and day 7 median and range of serum levels for total SARS-CoV-2 antibody and for IgM and IgG SARS-CoV-2 spike trimer antibody levels are shown. All comparisons by Wilcoxon Rank Sum Test at baseline, day 2, and day 7 between zanubrutinib- and placebo-treated patients were not significant.

| **Antibody** | **Arm** | **Baseline** | **Day 2** | **Day 7** |
| --- | --- | --- | --- | --- |
| SARS.S1 | Cohort 1 placebo | 1.07 (0.31-13.65) | 1.95 (0.54-11.76) | 10.58 (0.47-15.37) |
|  | Cohort 1 zanubrutinib | 1.68 (0.61-6.06) | 2.5 (0.91-9.21) | 5.89 (1.78-36.25) |
|  | Cohort 2 | 20.98 (4.5-114.5) | 22.32 (7.3-98.15) | 23.05 (11.41-88.2) |
|  |  |  |  |  |
| SARS.CoV-2  S1 protein | Cohort 1 placebo | 3.57 (0.16-366.63) | 20.55 (0.2-361.87) | 318.50 (1.38-464.27) |
|  | Cohort 1 zanubrutinib | 6.17 (1.01-247.16) | 31.82 (0.94-456.77) | 143.9 (3.94-720.83) |
|  | Cohort 2 | 480.25 (157.18-873.42) | 497.12 (317.11-720.16) | 585.19 (394.62-665.22) |
|  |  |  |  |  |
| SARS.CoV-2  spike trimer | Cohort 1 placebo | 3.57 (0.16-366.63) | 20.55 (0.2-361.87) | 318.50 (1.38-464.27) |
|  | Cohort 1 zanubrutinib | 31.18 (0.62-331.55) | 85.11 (0.59-673.74) | 410.85 (5.13-627.29) |
|  | Cohort 2 | 701.91 (239.25-917.32) | 624.02 (420.51-991.74) | 644.8 (486.81-1098.84) |
|  |  |  |  |  |
| SARS.CoV-2  RBD | Cohort 1 placebo | 6.83 (0.28-414.22) | 33.49 (0.46-741.51) | 316.07 (1.88-662.61) |
|  | Cohort 1 zanubrutinib | 11.77 (0.7-408.23) | 50.29 (0.64-451.41) | 178.29 (6.56-533.56) |
|  | Cohort 2 | 643.38 (229.05-818.19) | 570.19 (422.13-853.43) | 651.24 (397.33-953.22) |
|  |  |  |  |  |
| SARS.CoV-2  nucleocapsid | Cohort 1 placebo | 14.56 (0.3-612.3) | 101.94 (0.29-980.97) | 451.71 (0.73-962.67) |
|  | Cohort 1 zanubrutinib | 17.67 (0.71-432.67) | 56.06 (0.71-973.57) | 261.58 (11.05-3019.82) |
|  | Cohort 2 | 347.61 (51.94-1713.31) | 303.83 (80.04-2054.86) | 278.59 (125.95-1986.08) |
|  |  |  |  |  |
| SARS.CoV-2  IgM spike trimer | Cohort 1 placebo | 24829.14 (579.76-1356647.00) | 54440.66 (2158.52-1095305.90) | 120266.79 (9215.23-1298114.77) |
|  | Cohort 1 zanubrutinib | 36831.25 (1355.19-539776.22) | 98633.01 (981.76-1042400.4) | 149568.85 (4065.61-556510.79) |
|  | Cohort 2 | 288591.96 (155715.25-428875.62) | 179093.02 (79522.77-676351.51) | 104709.08 (41054.03-969662.25) |
|  |  |  |  |  |
| SARS.CoV-2  IgG spike trimer | Cohort 1 placebo | 356851.76 (7653.44-21437717.19) | 2251002.76 (8620.55-34857493.04) | 20008903.73 (28175.73-31632425.29) |
|  | Cohort 1 zanubrutinib | 642512.85 (12078.09-12530599.3) | 2656489.50 (9668.70-32410462.39) | 12324159.53 (202454.61-45248463.05) |
|  | Cohort 2 | 20241863.97 (10419950.99-43042871.69) | 30525778.97 (21763671.83-38625775.39) | 25797673.11 (15275457.96-38175932.16) |

Ig, immunoglobulin; RBD, receptor binding domain; SARS.CoV-2, severe acute respiratory syndrome coronavirus 2; S1, spike 1.

## **Supplementary Table 3. Baseline Cytokine and Chemokine Levels for Patients in Cohort 1 and 2.**

Baseline median and range of cytokine and chemokine levels are shown. Patients in cohort 2 trended or showed significantly lower levels of GM-CSF (p=0.09), IL-2 (p=0.029), IL-10 (p=0.06), IL-17A (p=0.069), and higher levels of IL-6 (p=0.012) and IL-8 (p=.009) relative to patients in cohort 1. Baseline cytokine levels between the two arms of cohort 1 were similar and showed no significant differences by Wilcoxon Rank Sum Test, with the exception of MCP-1 which was lower in zanubrutinib vs placebo treated patients at baseline (p=0.034).

| **Cytokine/chemokine** | **Cohort 1** | | **Cohort 2** |
| --- | --- | --- | --- |
|  | **Placebo** | **Zanubrutinib** | **Zanubrutinib** |
| G-CSF | 11.045 (4.35-30.1) | 9.3 (4.11-24.77) | 13.37 (10.48-17.75) |
| GM-CSF | 29 (1.95-166.23) | 23.27 (1.95-68.34) | **14.32 (6.42-16.02)** |
| IFN-α | 0.69 (0.21-57.33) | 0.51 (0.27-13.59) | NA^a^ |
| IFN-γ | 9.125 (3.48-35.6) | 6.98 (1.34-14.43) | 8.125 (4.5-13.68) |
| IL-1β | 8.075 (1.47-27.67) | 7.97 (1.22-34.82) | 7.845 (3.02-10.74) |
| IL-2 | 10.89 (1.69-120.35) | 8.2 (4.25-28.42) | **3.57 (2.84-6.19)** |
| IL-4 | 24.08 (5.62-49.35) | 21.275 (8.16-42.33) | 26.025 (11.69-36.93) |
| IL-5 | 7.29 (3.38-14.94) | 6.545 (6.25-6.84) | NA^a^ |
| IL-6 | 2 (0.12-53.83) | 0.57 (0.26-2.9) | **10.45 (9.9-13.4)** |
| IL-8 | 0.15 (0.03-0.92) | 0.14 (0.04-0.96) | **5.06 (0.75-9.11)** |
| IL-10 | 2.2 (0.24-12.55) | 1.66 (0.49-22.97) | **0.27 (0.25-2.03)** |
| IL-12p70 | 10.215 (2.95-69.05) | 7.27 (3.45-24.84) | 5.1 (3.14-7.06) |
| IL-13 | 6.23 (1.09-36.52) | 4.36 (1.62-417.7) | 2.42 (2.42-2.42) |
| IL-17A | 1.76 (0.5-40.98) | 1.34 (0.45-7.55) | **0.74 (0.49-1.28)** |
| IL-18 | 45.44 (6.4-181.78) | 46.035 (13.64-146.23) | 58.37 (43.15-64.85) |
| IP-10/CXCL10 | 89.615 (3.6-493.59) | 89.61 (5.41-945.4) | 92.43 (47-195.67) |
| MCP-1 | **49.62 (3.27-869.42)** | 30.135 (4.57-222.13) | 117.53 (34.79-198.35) |
| MIP-1α | 7.76 (0.96-72.23) | 4.2 (0.85-87.09) | 3.39 (0.56-5.97) |
| MIP-1β | 11.065 (0.85-142.26) | 20.815 (0.03-61.63) | 17.835 (14.99-54.8) |
| TNF-α | 5.48 (0.61-47.08) | 3.42 (1.45-11.5) | 2.275 (0.71-3.21) |

**Red bold** text indicates statistical significance between cohort 1 and cohort 2.

**Black bold** text indicates statistical significance between placebo and zanubrutinib arms in cohort 1.

^a^NA denotes median values not available for that cohort.
G-CSF, granulocyte colony-stimulating factor; GM-CSF, granulocyte-macrophage colony-stimulating factor; IFN, interferon; MCP, monocyte chemoattractant protein; MIP, macrophage inflammatory protein; TNF, tumor necrosis factor.

## **Supplementary Table 4. Baseline and Serial Cytokine and Chemokine Levels for Patients in Cohort 1 and 2.**

Median and range of baseline and serial cytokine/chemokine levels obtained on days 2 and 7 are shown. Following treatment, lower levels for G-CSF, IL-10, and MCP-1 were observed on both days 2 and 7 (p<0.05 on both days) and for IL-4 and IL-13 on day 7 (p<0.05) by Wilcoxon Rank Sum Test, though levels for MCP-1 were lower at baseline (see Supplementary Table 3; p=0.034). Following FDR correction, these findings were statistically unremarkable.

| **Cytokine** | **Arm** | **Baseline** | **Day 2** | **Day 7** |
| --- | --- | --- | --- | --- |
| G-CSF | Cohort 1 placebo | 11.045 (4.35-30.1) | 10.03 (5.67-33) | 13.66 (4.42-29.69) |
|  | Cohort 1 zanubrutinib | 9.3 (4.11-24.77) | **7.625 (3-26.65)** | **5.76 (4.61-17.34)** |
|  | Cohort 2 | 13.37 (10.48-17.75) | 10.87 (10.48-14.06) | 12.1 (7.41-15.83) |
|  |  |  |  |  |
| GM-CSF | Cohort 1 placebo | 29 (1.95-166.23) | 26.9 (3.19-796.24) | 47.08 (2.13-76.1) |
|  | Cohort 1 zanubrutinib | 23.27 (1.95-68.34) | 17.57 (4.91-48.87) | 31.635 (18.74-55.89) |
|  | Cohort 2 | 14.32 (6.42-16.02) | 9.8 (3.19-11.49) | 17.72 (7.61-21.11) |
|  |  |  |  |  |
| IFN-α | Cohort 1 placebo | 0.69 (0.21-57.33) | 6.225 (2.22-12.91) | NA^a^ |
|  | Cohort 1 zanubrutinib | 0.51 (0.27-13.59) | 0.37 (0.37-0.37) | NA^a^ |
|  | Cohort 2 | NA^a^ | NA^a^ | NA^a^ |
|  |  |  |  |  |
| IFN-γ | Cohort 1 placebo | 9.125 (3.48-35.6) | 7.23 (0.89-118.97) | 10.84 (1.95-78.94) |
|  | Cohort 1 zanubrutinib | 6.98 (1.34-14.43) | 6.4 (2.25-13.04) | 6.33 (1.34-12.77) |
|  | Cohort 2 | 8.125 (4.5-13.68) | 5.99 (3.23-10.22) | 9.015 (4.45-27.45) |
|  |  |  |  |  |
| IL-1β | Cohort 1 placebo | 8.075 (1.47-27.67) | 6.96 (2.33-152.31) | 11.57 (3.02-31.49) |
|  | Cohort 1 zanubrutinib | 7.97 (1.22-34.82) | 7.12 (2.02-20.82) | 5.49 (1.88-28.93) |
|  | Cohort 2 | 7.845 (3.02-10.74) | 3.975 (0.63-4.77) | 4.86 (2.66-11.44) |
|  |  |  |  |  |
| IL-2 | Cohort 1 placebo | 10.89 (1.69-120.35) | 9.61 (1.53-328.25) | 20.78 (6.19-54.56) |
|  | Cohort 1 zanubrutinib | 8.2 (4.25-28.42) | 7.355 (3.34-19.99) | 8.3 (7.28-15.64) |
|  | Cohort 2 | 3.57 (2.84-6.19) | 3.81 (3.81-3.81) | 7.56 (7.11-8.01) |
|  |  |  |  |  |
| IL-4 | Cohort 1 placebo | 24.08 (5.62-49.35) | 16.49 (4.39-145.24) | 19.52 (12.2-38.76) |
|  | Cohort 1 zanubrutinib | 21.275 (8.16-42.33) | 14.405 (5.1-27.34) | **9.68 (4.42-28.77)** |
|  | Cohort 2 | 26.025 (11.69-36.93) | 23.055 (8.19-26.39) | 21.375 (11.24-34.47) |
|  |  |  |  |  |
| IL-5 | Cohort 1 placebo | 7.29 (3.38-14.94) | 97.27 (97.27-97.27) | 4.68 (4.68-4.68) |
|  | Cohort 1 zanubrutinib | 6.545 (6.25-6.84) | NA^a^ | NA^a^ |
|  |  |  |  |  |
| IL-6 | Cohort 1 placebo | 2 (0.12-53.83) | 0.85 (0.32-65.95) | 11.59 (3.08-98.77) |
|  | Cohort 1 zanubrutinib | 0.57 (0.26-2.9) | 1.78 (0.24-8.96) | 0.75 (0.21-62.49) |
|  | Cohort 2 | 10.45 (9.9-13.4) | 13.25 (9.93-16.57) | 6.93 (1.44-58.14) |
|  |  |  |  |  |
| IL-8 | Cohort 1 placebo | 0.15 (0.03-0.92) | 0.2 (0.08-1.8) | 1.935 (0.08-5.94) |
|  | Cohort 1 zanubrutinib | 0.14 (0.04-0.96) | 0.16 (0.08-0.52) | 0.39 (0.21-1.82) |
|  | Cohort 2 | 5.06 (0.75-9.11) | 7.4 (6.39-8.41) | 26.29 (14.21-38.37) |
|  |  |  |  |  |
| IL-10 | Cohort 1 placebo | 2.2 (0.24-12.55) | 2.36 (0.31-26.42) | 3.315 (1.17-12.98) |
|  | Cohort 1 zanubrutinib | 1.66 (0.49-22.97) | **0.74 (0.22-6.74)** | **0.67 (0.41-2.25)** |
|  | Cohort 2 | 0.27 (0.25-2.03) | 0.91 (0.63-2.37) | 0.505 (0.22-0.66) |
|  |  |  |  |  |
| IL-12p70 | Cohort 1 placebo | 10.215 (2.95-69.05) | 7.36 (1.79-300.41) | 19.935 (5.63-52.97) |
|  | Cohort 1 zanubrutinib | 7.27 (3.45-24.84) | 5.69 (3.4-15.4) | 5.295 (2.95-11.43) |
|  | Cohort 2 | 5.1 (3.14-7.06) | 3.575 (3.14-4.01) | 6.19 (4.01-7.06) |
|  |  |  |  |  |
| IL-13 | Cohort 1 placebo | 6.23 (1.09-36.52) | 5.26 (1.09-103.03) | 7.605 (2.43-25.46) |
|  | Cohort 1 zanubrutinib | 4.36 (1.62-417.7) | 3.515 (1.98-361.41) | **1.93 (1.38-3.64)** |
|  | Cohort 2 | 2.42 (2.42-2.42) | NA^a^ | 2.42 (2.11-3.92) |
|  |  |  |  |  |
| IL-17A | Cohort 1 placebo | 1.76 (0.5-40.98) | 1.44 (0.41-76.84) | 1.515 (0.77-17.26) |
|  | Cohort 1 zanubrutinib | 1.34 (0.45-7.55) | 1.42 (0.38-9.49) | 1.435 (0.22-2.81) |
|  | Cohort 2 | 0.74 (0.49-1.28) | 0.65 (0.65-0.65) | 1 (0.37-1.05) |
|  |  |  |  |  |
| IP-10/CXCL10 | Cohort 1 placebo | 89.62 (3.6-493.55) | 54.88 (19.01-391.76) | 99.77 (9.25-504.62) |
|  | Cohort 1 zanubrutinib | 89.61 (5.41-945.4) | 70.44 (8.96-556.86) | 49 (17.22-364.08) |
|  | Cohort 2 | 92.43 (47-195.67) | 94.23 (42.24-98.71) | 105.13 (23.85-246.69) |
|  |  |  |  |  |
| MCP-1 | Cohort 1 placebo | 49.62 (3.27-869.42) | 70.99 (5.61-1420.13) | 66.8 (26.22-217.5) |
|  | Cohort 1 zanubrutinib | 30.135 (4.57-222.13) | 34.465 (5.35-147.81) | 34.4 (7.63-616.72) |
|  | Cohort 2 | 117.53 (34.79-198.35) | 55.77 (32.4-134.1) | 124.01 (34.5-171.4) |
|  |  |  |  |  |
| MIP-1α | Cohort 1 placebo | 7.76 (0.96-72.23) | 5.525 (0.4-72.45) | 4.71 (0.95-69.89) |
|  | Cohort 1 zanubrutinib | 4.2 (0.85-87.09) | 4.12 (0.4-106.29) | 2.405 (0.25-24.49) |
|  | Cohort 2 | 3.39 (0.56-5.97) | 1.86 (1.07-4.16) | 4.22 (2.97-5.78) |
|  |  |  |  |  |
| MIP-1β | Cohort 1 placebo | 11.065 (0.85-142.26) | 11.55 (1.42-71.72) | 14.73 (0.48-70.85) |
|  | Cohort 1 zanubrutinib | 20.815 (0.03-61.63) | 15.605 (0.64-55.44) | 16.345 (2.83-41.5) |
|  | Cohort 2 | 17.835 (14.99-54.8) | 17.955 (9.19-23.87) | 39.1 (29.52-47.97) |
|  |  |  |  |  |
| IL-18 | Cohort 1 placebo | 45.44 (6.4-181.78) | 43.31 (22.11-233.73) | 54.97 (41.43-979.53) |
|  | Cohort 1 zanubrutinib | 46.035 (13.64-146.23) | 45.155 (13.39-119.57) | 32.67 (7.73-73.29) |
|  | Cohort 2 | 58.37 (43.15-64.85) | 48.32 (38.77-62.24) | 87.21 (40.98-139.75) |
|  |  |  |  |  |
| TNF-α | Cohort 1 placebo | 5.48 (0.61-47.08) | 3.74 (0.85-161.31) | 12.37 (5.4-23.15) |
|  | Cohort 1 zanubrutinib | 3.42 (1.45-11.5) | 3.84 (0.85-9.55) | 1.83 (1.66-20.34) |
|  | Cohort 2 | 2.275 (0.71-3.21) | 2.11 (0.71-3.51) | 3.345 (1.5-8.94) |
|  |  |  |  |  |
| TNF-β | Cohort 1 placebo | 18.56 (8.46-262.04) | 141.59 (73.92-217.71) | 145.81 (145.81-145.81) |
|  | Cohort 1 zanubrutinib | 230.55 (230.55-230.55) | 210.26 (210.26-210.26) | NA^a^ |
|  | Cohort 2 | NA^a^ | NA^a^ | NA^a^ |

**Red bold** text indicates p<0.05

^a^NA denotes median values not available for that cohort.

FDR, false discovery rate; G-CSF, granulocyte colony-stimulating factor; GM-CSF, granulocyte-macrophage colony-stimulating factor; IFN, interferon; IL, interleukin; MCP, monocyte chemoattractant protein; TNF, tumor necrosis factor.

## **Supplementary Table 5. Findings From Single-Cell Transcriptome Studies for Cohort 1 Patients.**

Peripheral blood mononuclear cells were used for these experiments; samples were obtained at pretreatment and at 2 days post-treatment. Differential gene expression analyses were performed using DESeq2 (v. 1.34.0). This was done by generating a pseudo-bulk count matrix for each cell type. Differences in cell type proportions between zanubrutinib and placebo were tested for each time point using both propeller from speckle (5) and a Wilcoxon test. Significant cytokine, chemokine, or signaling pathway gene of interest are shown, along with their relative upregulation or downregulation in context of specific immune effector cell type.

| **Gene** | **Group** | **Cell Type** | **Change** | **Log2fc** | **p-value** | **Significance** |
| --- | --- | --- | --- | --- | --- | --- |
| IL-6 | Zanubrutinib | Naive B cells | ↓ | −1.20 | 0.030 | Significant |
|  | Placebo | Naive B cells |  | 0.62 | 0.079 | Non-significant |
|  | Zanubrutinib | Memory B cells | ↓ | −2.61 | 0.030 | Significant |
|  | Placebo | Memory B cells |  | −0.32 | 0.835 | Non-significant |
|  |  |  |  |  |  |  |
| IL-7 | Zanubrutinib | Naive B cells | ↑ | 1.30 | 0.000 | Significant |
|  | Placebo | Naive B cells |  | 0.49 | 0.177 | Non-significant |
|  |  |  |  |  |  |  |
| IL-8 | Zanubrutinib | CD14 monocytes | ↓ | −0.86 | 0.009 | Significant |
|  | Placebo | CD14 monocytes |  | 0.53 | 0.367 | Non-significant |
|  |  |  |  |  |  |  |
| CSF1/M-CSF | Zanubrutinib | CD14 monocytes | ↓ | −1.80 | 0.029 | Significant |
|  | Placebo | CD14 monocytes |  | −0.36 | 0.571 | Non-significant |
|  | Zanubrutinib | CD4 effector memory T cells |  | 1.20 | 0.327 | Non-significant |
|  | Placebo | CD4 effector memory T cells | ↑ | 2.60 | 0.032 | Significant |
|  |  |  |  |  |  |  |
| CXCR4 | Zanubrutinib | CD14 monocytes | ↓ | −1.46 | 0.001 | Significant |
|  | Placebo | CD14 monocytes |  | −0.67 | 0.083 | Non-significant |
|  | Zanubrutinib | CD8 naive T cells | ↓ | −0.47 | 0.001 | Significant |
|  | Placebo | CD8 naive T cells |  | −0.28 | 0.176 | Non-significant |
|  | Zanubrutinib | CD4 proliferating T cells | ↓ | −1.40 | 0.000 | Significant |
|  | Placebo | CD4 proliferating T cells |  | −1.39 | 0.071 | Non-significant |
|  | Zanubrutinib | CD4 naive T cells | ↓ | −0.50 | 0.007 | Significant |
|  | Placebo | CD4 naive T cells | ↓ | −0.60 | 0.010 | Significant |
|  | Zanubrutinib | CD56^bright^ NK cells | ↓ | −1.00 | 0.010 | Significant |
|  | Placebo | CD56^bright^ NK cells |  | −0.49 | 0.361 | Non-significant |
|  | Zanubrutinib | CD16 monocytes | ↓ | −1.40 | 0.006 | Significant |
|  | Placebo | CD16 monocytes |  | −0.97 | 0.076 | Non-significant |
|  | Zanubrutinib | CD8 central memory T cells | ↓ | −0.30 | 0.010 | Significant |
|  | Placebo | CD8 central memory T cells |  | −0.29 | 0.185 | Non-significant |
|  | Zanubrutinib | Hematopoietic stem and  progenitor cell | ↓ | −0.90 | 0.030 | Significant |
|  | Placebo | Hematopoietic stem and  progenitor cell |  | −0.85 | 0.471 | Non-significant |
|  | Placebo | Gamma delta T cells | ↓ | −0.60 | 0.007 | Significant |
|  | Zanubrutinib | Gamma delta T cells |  | −0.33 | 0.191 | Non-significant |
|  | Placebo | Memory B cells | ↓ | −0.40 | 0.030 | Significant |
|  | Zanubrutinib | Memory B cells |  | 0.20 | 0.386 | Non-significant |
|  | Placebo | Plasmablasts | ↓ | −0.80 | 0.020 | Significant |
|  | Zanubrutinib | Plasmablasts |  | −0.47 | 0.139 | Non-significant |
|  | Placebo | CD8 effector memory T cells | ↓ | −0.60 | 0.030 | Significant |
|  | Zanubrutinib | CD8 effector memory T cells |  | −0.11 | 0.564 | Non-significant |
|  |  |  |  |  |  |  |
| MIP-1α | Zanubrutinib | CD14 monocytes | ↓ | −2.07 | 0.001 | Significant |
|  | Placebo | CD14 monocytes | ↑ | 1.04 | 0.056 | Non-significant |
|  | Zanubrutinib | Platelets | ↓ | −2.12 | 0.003 | Significant |
|  | Placebo | Platelets |  | 0.40 | 0.635 | Non-significant |
|  | Zanubrutinib | CD4 effector memory T cells | ↓ | −3.76 | 0.007 | Significant |
|  | Placebo | CD4 effector memory T cells |  | −0.88 | 0.786 | Non-significant |
|  | Zanubrutinib | Plasmablasts | ↓ | −2.32 | 0.002 | Significant |
|  | Placebo | Plasmablasts |  | −1.39 | 0.265 | Non-significant |
|  |  |  |  |  |  |  |
| IL-1β | Zanubrutinib | CD14 monocytes | ↓ | −1.19 | 0.001 | Significant |
|  | Placebo | CD14 monocytes |  | 1.14 | 0.109 | Non-significant |
|  |  |  |  |  |  |  |
| IFN-γ | Zanubrutinib | Gamma delta T cells | ↑ | 2.09 | 0.012 | Significant |
|  | Placebo | Gamma delta T cells |  | −0.21 | 0.855 | Non-significant |
|  |  |  |  |  |  |  |
| TNF-α | Zanubrutinib | CD14 monocytes |  | −0.16 | 0.568 | Non-significant |
|  | Placebo | CD14 monocytes | ↑ | 0.74 | 0.036 | Significant |
|  |  |  |  |  |  |  |
| IFNAR1 | Zanubrutinib | CD14 monocytes | ↓ | 0.28 | 0.035 | Significant |
|  | Placebo | CD14 monocytes |  | 0.18 | 0.188 | Non-significant |
|  |  |  |  |  |  |  |
| IFNAR2 | Zanubrutinib | CD4 central memory T cells | ↓ | 0.22 | 0.044 | Significant |
|  | Placebo | CD4 central memory T cells |  | 0.10 | 0.499 | Non-significant |
|  |  |  |  |  |  |  |
| JAK1 | Zanubrutinib | CD14 monocytes | ↓ | 0.21 | 0.007 | Significant |
|  | Placebo | CD14 monocytes |  | 0.04 | 0.814 | Non-significant |
|  |  |  |  |  |  |  |
| JAK3 | Zanubrutinib | CD14 monocytes | ↓ | 0.62 | 0.017 | Significant |
|  | Placebo | CD14 monocytes |  | 0.35 | 0.160 | Non-significant |
|  | Zanubrutinib | CD4 naive T cells | ↓ | −0.48 | 0.039 | Significant |
|  | Placebo | CD4 naive T cells |  | 0.20 | 0.466 | Non-significant |
|  |  |  |  |  |  |  |
| TYK2 | Zanubrutinib | CD4 central memory T cells | ↓ | −0.38 | 0.019 | Significant |
|  | Placebo | CD4 central memory T cells |  | 0.07 | 0.744 | Non-significant |
|  | Zanubrutinib | Memory B cells | ↓ | −0.84 | 0.038 | Significant |
|  | Placebo | Memory B cells |  | 0.25 | 0.606 | Non-significant |
|  | Zanubrutinib | CD4 Effector Memory T cells | ↓ | −0.86 | 0.016 | Significant |
|  | Placebo | CD4 effector memory T cells |  | −0.49 | 0.313 | Non-significant |
|  | Placebo | CD8 effector memory T cells | ↓ | −0.53 | 0.033 | Significant |
|  | Zanubrutinib | CD8 effector memory T cells |  | −0.07 | 0.788 | Non-significant |
|  |  |  |  |  |  |  |
| STAT2 | Zanubrutinib | CD4 central memory T cells | ↓ | −0.54 | 0.002 | Significant |
|  | Placebo | CD4 central memory T cells |  | −0.23 | 0.262 | Non-significant |
|  |  |  |  |  |  |  |
| STAT3 | Zanubrutinib | CD14 monocytes | ↓ | 0.38 | 0.046 | Significant |
|  | Placebo | CD14 monocytes |  | 0.15 | 0.389 | Non-significant |
|  | Zanubrutinib | Platelets | ↓ | 0.67 | 0.022 | Significant |
|  | Placebo | Platelets |  | 0.03 | 0.923 | Non-significant |
|  |  |  |  |  |  |  |
| MIP-3α | Zanubrutinib | CD14 monocytes | ↓ | −3.06 | 0.000 | Significant |
|  | Placebo | CD14 monocytes |  | −0.80 | 0.190 | Non-significant |

CSF, colony-stimulating factor; CXCR, C-X-C chemokine receptor; IFN, interferon; IFNAR, interferon alpha and beta receptor; IL, interleukin; JAK, Janus kinase; M-CSF, macrophage colony-stimulating factor; MIP, macrophage inflammatory protein; NK, natural killer; STAT, signal transducer and activator of transcription; TNF, tumor necrosis factor; TYK, tyrosine kinase.

# Supplementary References

1. Cao B, Wang Y, Wen D, Liu W, Wang J, Fan G, et al. A trial of lopinavir-ritonavir in adults hospitalized with severe Covid-19. *N Engl J Med* (2020) 382(19):1787–99. doi: 10.1056/NEJMoa2001282

2. Richardson S, Hirsch JS, Narasimhan M, Crawford JM, McGinn T, Davidson KW, et al. Presenting characteristics, comorbidities, and outcomes among 5700 patients hospitalized with COVID-19 in the New York city area. *JAMA* (2020) 323(20):2052–9. doi: 10.1001/jama.2020.6775

3. Stuart T, Butler A, Hoffman P, Hafemeister C, Papalexi E, Mauck WM, 3rd, et al. Comprehensive integration of single-cell data. *Cell* (2019) 177(7):1888–902. doi: 10.1016/j.cell.2019.05.031

4. Hao Y, Hao S, Andersen-Nissen E, Mauck WM, 3rd, Zheng S, Butler A, et al. Integrated analysis of multimodal single-cell data. *Cell* (2021) 184(13):3573–87. doi: 10.1016/j.cell.2021.04.048

5. Phipson B, Sim CB, Porrello ER, Hewitt AW, Powell J, Oshlack A. Propeller: testing for differences in cell type proportions in single cell data. *Bioinformatics* (2022) 38(20):4720–6. doi: 10.1093/bioinformatics/btac582
